# Development of Chinese Adolescents: Assessment, Issues, and Intervention

**DOI:** 10.1100/2012/935096

**Published:** 2012-09-03

**Authors:** Daniel T. L. Shek, Rachel C. F. Sun, Joav Merrick

**Affiliations:** ^1^Department of Applied Social Sciences, The Hong Kong Polytechnic University, Hong Kong; ^2^Public Policy Research Institute, The Hong Kong Polytechnic University, Hong Kong; ^3^Department of Social Work, East China Normal University, Shanghai 200062, China; ^4^Kiang Wu Nursing College of Macau, Macau; ^5^Division of Adolescent Medicine, Department of Pediatrics, Kentucky Children's Hospital, University of Kentucky College of Medicine, Lexington, KY 40506, USA; ^6^Faculty of Education, The University of Hong Kong, Hong Kong; ^7^National Institute of Child Health and Human Development, 91012 Jerusalem, Israel

In many places, young are regarded as future assets of the society. Hence, adolescent prevention programs are commonly developed to tackle adolescent risk behavior and positive youth development programs are designed to promote holistic development in adolescents [1]. However, a survey of the literature shows that research on adolescents is mainly confined to the western societies. Using the search term “adolescence,” computer search conducted in October 2011 showed that there were 299,266 citations in PsycINFO, 11,135 citations in ERIC, 54,313 citations in MEDLINE, and 48,045 citations in CINAHL. However, when using the search terms “Chinese” and “adolescence,” computer search showed that there were 4,881 citations in PsycINFO, 108 citations in ERIC, 374 citations in MEDLINE, and 398 citations in CINAHL. Similar phenomena were observed when “assessment,” “risk behavior,” and “prevention” as search terms were added (see Table 1).

For Chinese psychologists, pediatricians, psychiatrists, and allied human service workers, knowledge about adolescent development is largely developed in the western culture. To what extent western knowledge on adolescent development is applicable to Chinese young people? Are Chinese adolescent risk behaviors similar to those in western societies? To what extent intervention programs, such as adolescent prevention programs, are applicable to Chinese people? These are important questions to be addressed by human service professionals working with Chinese adolescents and their families.

There are three possible responses to cross-cultural variations in adolescent risk behavior, assessment, and intervention programs. First, based on the premise of cultural universalism (adolescent behavior is invariant across cultures), some colleagues may simply apply western knowledge on adolescents in Chinese contexts without much cultural adaptation. However, this approach has been criticized because the western culture is more “individualistic” whereas the Chinese culture is more “collectivistic.” Second, for those who believe that human behavior is not invariant in different cultures (i.e., cultural relativism), they argue that it is important to develop indigenous adolescent development knowledge, assessment tools, and intervention programs. However, in doing so, rigorous research is needed. Third, for those who hold a middle-of-the-road stand, they may argue that it is necessary to integrate both western and local knowledge and concepts. Obviously, irrespective of which approach one adopts, rigorous research on adolescent development, assessment, and intervention plays an important role in the process.

Regarding cross-cultural variations on adolescent development, three issues deserve special attention. The first issue is assessment of adolescent behavior. According to Hudson [2], there are two axioms of treatment—The first states: “If you cannot measure the client's problem, it does not exist.” The second, a corollary of the first, states: “If you cannot measure the client's problem, you cannot treat it” (p.65). In the realm of social sciences, assessment is the “soul” of research because it helps to operationalize the abstract theoretical constructs in the reality. It is also the essence of positivism and postpositivism. Unfortunately, as pointed out by Shek [3], objective psychosocial assessment tools on children and adolescents in different Chinese communities are scarce. Therefore, more research on developing valid and reliable assessment tools for adolescent behavior is of paramount importance.

The second area that deserves attention is adolescent risk behavior such as substance abuse, mental health problems, violence, and school dropout. In many western countries, there are established research programs with periodic charting of adolescent risk behavior, such as Monitoring the Future and National Survey on Drug Use and Health. Besides, numerous longitudinal studies have been conducted in the west to understand risk behavior in adolescents in western societies. In such studies, besides showing the prevalence and psychosocial correlates of adolescent risk behavior, risk factors that increase the probability of adolescent risk behavior are also identified. In the western literature, there are research studies showing that risk factors on the individual (e.g., hopelessness), family (e.g., habitual family conflicts), interpersonal (e.g., social alienation), community (e.g., poverty culture), and societal (e.g., unemployment) levels would lead to an increased probability of adolescent risk behavior. From a cross-cultural perspective, negative cultural beliefs such as pessimism and parenting characteristics such as overcontrol of children are risk factors for adolescent problem behavior.

The final area that deserves our attention is on the development of validated adolescent prevention and positive youth development programs for Chinese adolescents. With the rising adolescent developmental issues in the western contexts, many adolescent prevention programs have been developed. For example, an examination of the website of the Substance Abuse and Mental Health Services Administration showed that there are many adolescent prevention programs in the United States and programs are classified into different types according to their scientific credibility (e.g., “promising” programs and “exemplary” programs). Such a database is very important as far as evidence-based adolescent intervention work is concerned. In contrast to the western picture, the situation in Asia and different Chinese contexts is comparatively disappointing. According to Shek and Yu [4], validated adolescent prevention and positive youth development programs are almost nonexistent in different Chinese contexts, with the notable exception of the Project P.A.T.H.S. in Hong Kong. Through the use of different evaluation mechanisms, longitudinal evaluation findings in the Project P.A.T.H.S. showed that (a) different stakeholders held positive views of the program, instructors, and effectiveness of the program and, (b) those who participated in the program had better holistic development but less problem behavior (including substance abuse, delinquency, and behavioral intention to engage in problem behavior) than did those who did not participate in the program [5–11]. Besides the positive evaluation findings, it is exciting to know that the Project P.A.T.H.S. has been implemented in other parts of China, including Shanghai and Macau. Furthermore, the positive youth development constructs adopted in the Project P.A.T.H.S. are also used in a “university version” of the Project P.A.T.H.S., with the first and second authors developing a subject entitled “Tomorrow's Leaders” at The Hong Kong Polytechnic University. The preliminary evaluation result of this subject is very encouraging [12–15]. In the long run, it is suggested that more adolescent prevention and positive youth development programs should be developed in different Chinese communities. Why is it so? The answer is simple because Chinese people constitute roughly one-fifth of the world's population. The sheer huge number speaks for itself.

## Figures and Tables

**Table 1:** Literature search results based on major databases.

Database and search terms	Search any where	
	All publication	Peer-reviewed journal
*PsycINFO (1806+)*		
“Adolescence”	299266	216357
“Chinese” and “adolescence”	4881	4367
“Adolescence” and “assessment”	34189	26207
“Chinese” and “adolescence” and “assessment”	449	412
“Adolescence” and “risk behavior”	20059	15672
“Chinese” and “adolescence” and “risk behavior”	218	197
“Adolescence” and “prevention”	31078	19570
“Chinese” and “adolescence” and “prevention”	355	287

*MEDLINE (2000+) via ProQuest*		
“Adolescence”	54313	3667
“Chinese” and “adolescence”	374	45
“Adolescence” and “assessment”	3874	428
“Chinese” and “adolescence” and “assessment”	23	2
“Adolescence” and “risk behavior”	4453	357
“Chinese” and “adolescence” and “risk behavior”	27	3
“Adolescence” and “prevention”	5572	280
“Chinese” and “adolescence” and “prevention”	42	5

*ERIC via ProQuest (1966+)*		
“Adolescence”	11135	4003
“Chinese” and “adolescence”	108	65
“Adolescence” and “assessment”	628	286
“Chinese” and “adolescence” and “assessment”	3	2
“Adolescence” and “risk behavior”	1008	603
“Chinese” and “adolescence” and “risk behavior”	4	3
“Adolescence” and “prevention”	710	324
“Chinese” and “adolescence” and “prevention”	0	0

*CINAHL (1982+)*		
“Adolescence”	48045	44972
“Chinese” and “adolescence”	398	395
“Adolescence” and “assessment”	5888	5785
“Chinese” and “adolescence” and “assessment”	54	54
“Adolescence” and “risk behavior”	1600	1520
“Chinese” and “adolescence” and “risk behavior”	16	16
“Adolescence” and “prevention”	8088	7366
“Chinese” and “adolescence” and “prevention”	37	37
